# Epidemiology of *Trypanosoma evansi* and *Trypanosoma vivax* in domestic animals from selected districts of Tigray and Afar regions, Northern Ethiopia

**DOI:** 10.1186/s13071-015-0818-1

**Published:** 2015-04-09

**Authors:** Hadush Birhanu, Regassa Fikru, Mussa Said, Weldu Kidane, Tadesse Gebrehiwot, Ashenafi Hagos, Tola Alemu, Tesfaye Dawit, Dirk Berkvens, Bruno Maria Goddeeris, Philippe Büscher

**Affiliations:** College of Veterinary Medicine, Mekelle University, P. O. Box 2084, Mekelle, Ethiopia; Department of Biosystems, KU Leuven, Faculty of Bioscience Engineering, Kasteelpark Arenberg 30, B-3001, Leuven, Belgium; Department of Biomedical Sciences, Institute of Tropical Medicine, Nationalestraat 155, Antwerp, Belgium; College of Veterinary Medicine and Agriculture, Addis Ababa University, P.O. Box 34, Bishoftu, Ethiopia; Department of Statistics, College of Natural and Computational Sciences, Mekelle University, P.O.Box 231, Mekelle, Ethiopia; School of Veterinary Medicine, Hawassa University, P.O. Box 05, Hawassa, Ethiopia

**Keywords:** *Trypanosoma evansi* type A, *Trypanosoma evansi* type B, Dromedary camels, Equines, Ruminants, Ethiopia

## Abstract

**Background:**

African animal trypanosomosis, transmitted cyclically by tsetse flies or mechanically by other biting flies, causes serious inflictions to livestock health. This study investigates the extent of non-tsetse transmitted animal trypanosomosis (NTTAT) by *Trypanosoma (T.) evansi* and *T. vivax* in domestic animals in the tsetse-free regions of Northern Ethiopia, Afar and Tigray.

**Methods:**

A cross sectional study was conducted on 754 dromedary camels, 493 cattle, 264 goats, 181 sheep, 84 donkeys, 25 horses and 10 mules. The microhaematocrit centrifugation technique was used as parasitological test. Plasma was collected for serodiagnosis with CATT/*T.evansi* and RoTat 1.2 immune trypanolysis (ITL) while buffy coat specimens were collected for molecular diagnosis with *T. evansi* type A specific RoTat 1.2 PCR, *T. evansi* type B specific EVAB PCR and *T. vivax* specific *Tv*PRAC PCR.

**Results:**

The parasitological prevalence was 4.7% in Tigray and 2.7% in Afar and significantly higher (z = 2.53, p = 0.011) in cattle (7.3%) than in the other hosts. Seroprevalence in CATT/*T.evansi* was 24.6% in Tigray and 13.9% in Afar and was significantly higher (z = 9.39, p < 0.001) in cattle (37.3%) than in the other hosts. On the other hand, seroprevalence assessed by ITL was only 1.9% suggesting cross reaction of CATT/*T.evansi* with *T. vivax* or other trypanosome infections. Molecular prevalence of *T. evansi* type A was 8.0% in Tigray and in Afar and varied from 28.0% in horses to 2.2% in sheep. It was also significantly higher (p < 0.001) in camel (11.7%) than in cattle (6.1%), donkey (6%), goat (3.8%), and sheep (2.2%). Four camels were positive for *T. evansi* type B. Molecular prevalence of *T. vivax* was 3.0% and was similar in Tigray and Afar. It didn’t differ significantly among the host species except that it was not detected in horses and mules.

**Conclusions:**

NTTAT caused by *T. vivax* and *T. evansi*, is an important threat to animal health in Tigray and Afar. For the first time, we confirm the presence of *T. evansi* type B in Ethiopian camels. Unexplained results obtained with the current diagnostic tests in bovines warrant particular efforts to isolate and characterise trypanosome strains that circulate in Northern Ethiopia.

## Background

Ethiopia is the richest country in livestock population in Africa with more than 52 million heads of cattle, 46 million small ruminants, about 9 million equines (donkeys, horses and mules) and 1 million camels [1]. The livestock resource contributes to 12% of the total gross domestic product (GDP) and over 45% of the agricultural GDP of Ethiopia. However, the benefit derived from livestock is far below its potential. Inadequate food supply, high disease prevalence, poor genetic resources and poor marketing are the main bottlenecks for the development of the livestock sector [2].

African trypanosomosis is one of the most important animal diseases encountered in all agro-ecological zones of the country and hinders the efforts made for food self-sufficiency [3]. African trypanosomosis is a general term for infections in many different hosts (man and his domestic animals and wild animals) caused by various trypanosome species with *Trypanosoma (T.) brucei*, *T. congolense, T. vivax, T. evansi* and *T. equiperdum* as the most important ones [4]. African animal trypanosomoses (AAT) cause serious inflictions to the health of livestock ranging from anaemia, loss of condition and emaciation, abortion, death etc. [5-10]. The trypanosomes responsible for AAT in Ethiopia are *T. vivax*, *T. congolense*, *T. brucei*, *T. evansi* and *T. equiperdum* [11].

*T. congolense* and *T. brucei* are exclusively found in the tsetse-infested areas of Ethiopia while *T. evansi* and *T. equiperdum* occur in the tsetse-free areas. *T. vivax* can be found in both tsetse-infested and tsetse-free areas except in the highlands, which are >2500 meter above sea level [11,12].

In Africa, *T. vivax* is transmitted both cyclically by *Glossina spp*. and mechanically by horse flies (*Tabanidae*) and stable flies (*Stomoxys sp.)*. It circulates in several species of ungulates including cattle, small ruminants, equids, camelids and wild animals such as antelopes [4]. Wild ungulates, especially buffaloes and antelopes, as well as trypanotolerant cattle are generally symptomless carriers [13]. *T. vivax* is also endemic in Latin America where its transmission is exclusively mechanical through biting flies [14-17].

*T. evansi* has multiple means of transmission of which mechanical transmission by biting insects is the most important in camels and other large animals. Other transmission routes such as the bite of vampire bats and oral transmission in carnivores has been documented [4,18,19].

In Ethiopia, *T. evansi* is widely distributed across the six agro-climatic zones and mainly coincides with the distribution of camels [20]. Trypanosomosis due to *T. evansi* (surra) is the number one protozoan disease of camels. Horses are also very susceptible. Infected camels and equines may die within 3 months. Moreover, cattle, buffalo, pigs, goat and sheep infected with *T. evansi* suffer from immunosuppression, resulting in increased susceptibility to other diseases or in vaccination failure [21-23]. For example, experimental infections in buffalo and pigs have shown reduced cellular and humoral responses after vaccination against classical swine fever and *Pasteurella multicoda* in *T. evansi* infected animals compared to uninfected animals [24-26].

*T. evansi* strains with kDNA minicircle type A are the most abundant and found in Africa, South America and Asia [27-29]. They are also characterised by the presence of the gene for the Variant Surface Glycoprotein (VSG) RoTat 1.2. This RoTat 1.2 VSG is expressed early during infections resulting in the detectability of anti-RoTat 1.2 antibodies in animals infected with *T. evansi* type A [30]. In contrast, *T. evansi* strains with type B minicircle are far less common and have so far been isolated only from camels in Kenya [31-35]. Ngaira *et al.* showed that *T. evansi* type B typically lacks the RoTat 1.2 gene and as a consequence, infections with this type are not detected with serological and molecular tests based on RoTat 1.2 VSG, like CATT/*T.evansi* and RoTat 1.2 PCR [32,34,36,37].

Despite the considerable number of epidemiological studies carried out in Ethiopia on cattle and camel trypanosomosis in parts of Southern Nations, Nationalities, and Peoples’ Region (SNNPR), and in Oromiya and Amhara regions, information from Tigray and pastoral areas of Afar, belonging to the tsetse-free areas of Ethiopia, is scanty [38-45]. In addition, due to limited logistic resources and poor diagnostic facilities, the exact burden and socioeconomic impact of AAT is probably underestimated and information on prevailing trypanosome species and affected hosts remains inaccurate and fragmented [44,46,47]. Therefore, this study was designed to investigate the distribution of *T. evansi* and *T. vivax* in selected districts of Tigray and in pastoral areas of Afar.

Diagnosis of AAT is often based on clinical suspicion. Parasite detection is cumbersome in many cases where only low numbers of trypanosomes circulate in the host body fluids [47]. Techniques for concentration of the trypanosomes by centrifugation of a blood specimen can be applied. After centrifugation of some blood in a capillary tube, the trypanosomes can be detected directly under the microscope at the level of the white blood cell layer (the buffy coat) [48]. Where differential diagnosis is needed, the capillary tube can be broken and the buffy coat spread on a microscope slide for examination according to Murray *et al.* [49]. A more sensitive technique is the mini Anion Exchange Centrifugation Technique (mAECT) but the technique works best with *T. brucei* and *T. evansi* and has poor diagnostic potential for *T. congolense* and *T. vivax* [50-53].

As an alternative to parasitological diagnosis, molecular diagnostic tests have been developed. For the diagnosis of surra, the PCR RoTat 1.2 and Q-PCR RoTat 1.2 are specific for *T. evansi* type A and PCR EVAB is specific for *T. evansi* type B [34,37,54]*.* For the molecular diagnosis of *T. vivax*, the ITS-1 PCR and proline racemase PCR (*Tv*PRAC PCR) can be employed [55,56]. Neither parasitological nor molecular tests are 100% sensitive, due to the often low number of circulating parasites.

Serological tests are able to reveal ongoing or past trypanosome infections based on antibody detection. For surra, the most specific antibody detection tests make use of the *T. evansi* specific variant surface glycoprotein (VSG) RoTat 1.2 as antigen. The CATT/*T.evansi* is such a test in the form of a direct agglutination test and is the only rapid diagnostic test for surra that is recommended by the World Organization for Animal Health [57,58]. By virtue of its format as a direct agglutination test, CATT/*T.evansi* can be applied on any host species. Knowledge about the antigenic repertoires of *T. vivax* is almost non-existent. Most antibody detection tests for *T. vivax* make use of more or less purified native antigens leaving room for non-specific reactions. In regions where *T. vivax* and *T. brucei* or *T. evansi* occur together in the same host species, it is almost impossible to identify the infecting trypanosome species at the level of circulating antibodies in the host [47,59-61]. Only recently, recombinant *T. vivax* specific antigens are being investigated for their diagnostic potential [62].

The present study provides data on the epidemiology of AAT in domestic animals in two tsetse-free regions of Ethiopia.

## Methods

### Study areas

**Figure 1:**
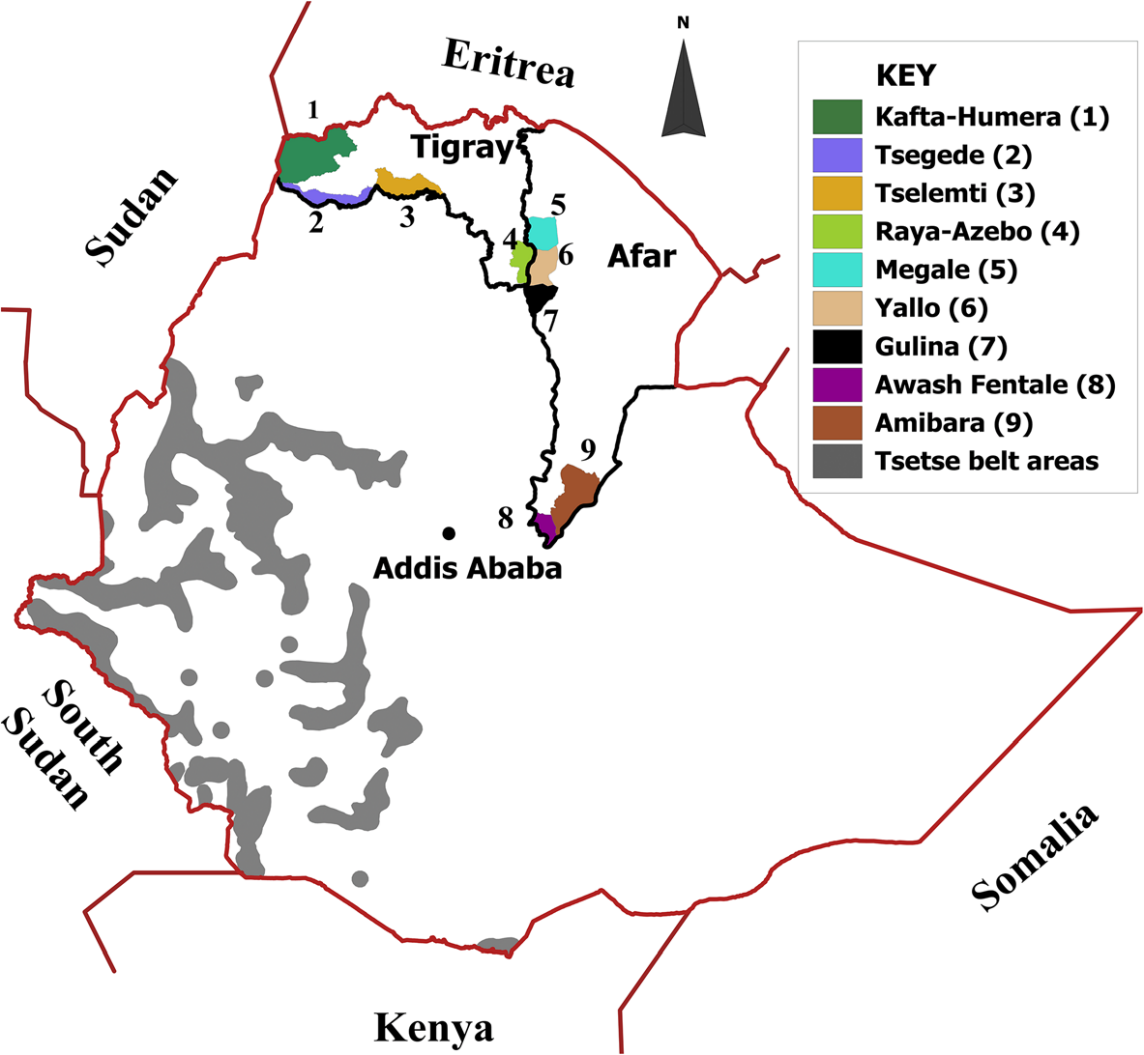
**Map of Ethiopia showing study districts in Tigray and Afar regions and tsetse belt areas.**

The study was conducted in selected districts (weredas) of Tigray and pastoral areas of Afar, representing tsetse-free areas of Ethiopia. Tigray region is located in the northern part of Ethiopia between longitudes 36°27′ E and 39°59′ E and latitudes 12°15′ N and 14°57′ N (Figure 1). It shares international boundaries with Eritrea and Sudan and regional boundaries with Amhara and Afar regions of Ethiopia. Tigray is divided into four zones and 35 weredas [63]. Selected “tabias” or peasant associations from the districts of Raya-Azebo (southern zone), Tselemti (northwestern zone) and Kafta-Humera and Tsegede (western zone), were included.

Afar region, one of the four major pastoral regions in Ethiopia, occupies an area of about 270,000 km^2^ and is situated between longitudes 39°34′E and 42°28′E and latitudes 8°49′N and 14°30′N [64]. The region shares international boundaries with the State of Eritrea and Djibouti, as well as regional boundaries with the regions of Tigray, Amhara, Oromia and Somali (Figure 1). The Afar region consists of 5 administrative zones (sub-regions) [65]. Taking into account the accessibility to the pastoral communities, “kebeles” or sampling stations were selected in the districts of Megale (zone 2), Awash Fentale and Amibara (zone 3) and Gulina and Yalo (zone 4).

### Ethics statement

The study protocol was approved by the Veterinary Ethics Committee of the Institute of Tropical Medicine (EXT2012-1).

### Study design, study animals and specimen collection

Considering 95% confidence level and average prevalence of 30% [44], the number of specimens to collect was planned according to Thrusfield [66] as n = (1.96)^2^ × P_exp_(1-P_exp_)/d^2^; where: n = required sample size, d = absolute precision required (d = 0.05), P_exp_ = expected prevalence of the disease. A cross sectional study was conducted from February till July 2013 on 1811 domestic animals comprising 754 dromedary camels, 493 cattle, 264 goats, 181 sheep, 84 donkeys, 25 horses and 10 mules. The animals were sampled at watering and grazing points and at veterinary clinics where they were brought for accaricide spraying and vaccination. Individual study subjects from willing owners were randomly selected regardless of age, gender and body condition [66]. From each animal, 9 mL of jugular vein blood was collected in a heparinised Venosafe tube (Terumo, Leuven, Belgium), labelled with a unique code, placed in a coolbox at 4°C and processed as described below.

### Packed cell volume (PCV) and microhaematocrit centrifugation technique (mHCT)

The microhaematocrit (mHCT) was performed as described by Woo [67]. Briefly, 4 microhaematocrit capillary tubes were filled with approximately 50 μL of blood from the Venosafe tube and stoppered with sealant. After centrifugation at 12,000 rpm for 5 min, the PCV was recorded and the tubes were mounted in a specially designed viewing chamber and examined under the microscope at 10x16 magnification for the presence of motile trypanosomes at the level of the buffy coat as described by Fikru *et al.* [44]. Animals with confirmed presence of trypanosomes were treated free of charge with 0.25 mg/kg melarsamine hydrochloride (cymelarsan) in the case of camels or with 0.5 mg/kg isometamidium chloride (samorin, trypamidium) or 7 mg/kg diminazeneaceturate (berenil) in the case of ruminants.

### Preparation of plasma and buffy coat specimens

The blood collected in the heparinised Venosafe tubes was centrifuged for 10 min at 1,000 rpm and plasma was collected with a single use plastic transfer pipette into 2 mL tubes with screwcaps (Sarstedt, Nümbrecht, Germany). Plasma was stored at 4°C until testing for specific antibodies with CATT/*T.evansi* and subsequently frozen at −20°C. From the remaining blood specimen, 500 μL of buffy coat layer were collected by means of a micropipette with a filter tip and mixed with an equal volume of guanidium EDTA buffer (GEB; 6 M guanidium chloride, 0.2 M EDTA, pH 8.0) and stored at ambient temperature until DNA extraction [68]. Of those animals that were parasitologically positive, part of the buffy coat was collected for cryopreservation in liquid nitrogen for later isolation of the parasite according to Pyana et al. [69].

### CATT/*T. evansi*

Detection of *T. evansi* specific antibodies was carried out by CATT/*T.evansi* on plasma that was prediluted 1:4 in CATT diluent, according to the instructions of the manufacturer (Institute of Tropical Medicine, Antwerp, Belgium).

### Immune trypanolysis test for surra

From each plasma specimen, 30 μL were spotted on Whatman 4 filter paper (Whatman, Maidstone, UK) in Ethiopia and shipped to the Institute of Tropical Medicine, Antwerp, Belgium. For elution of plasma and test procedures, the protocol developed by Camara and co-workers, with modifications, was employed [70]. Briefly, from each filter paper, two 6 mm diameter disks were punched and placed in a well of a flat bottom microlon microtitre plate (Greiner Bio-One, Wemmel, Belgium). Antibodies were eluted overnight at 4°C in 40 μL of fetal bovine serum (FBS) followed by 1 hour on a plate shaker at ambient temperature. Twenty μL of the eluted fraction were transferred into a well of a U-bottom polystyrene microtitre plate (Sterilin, Newport, UK). Next, *T. evansi* RoTat 1.2 trypanosomes, grown in a mouse, were diluted in ice-cold guinea pig serum (GPS) and kept on ice to obtain a suspension of 5 trypanosomes per microscopic field according to the matching method [71]. Twenty μL of this suspension were added to each well of the microtiter plate with the eluted specimens and incubated at ambient temperature for 1 hour. Antibody mediated complement lysis was assessed by dispensing 5 μL of the reaction mixture on a microscope slide, covered by a 18×18 mm cover slip and examination at 25×10 magnification under a phase-contrast microscope. Trypanolysis was considered positive when at least 50% of the trypanosomes were lysed [70].

### DNA extraction

DNA extraction was performed with the High Pure PCR Product Purification Kit (Roche Diagnostics, Mannheim, Germany). Since unexpected clotting of the buffy coat specimens preserved in GEB was observed, 200 μL of tissue lysis buffer and 50 μL of proteinase K (Roche Diagnostics, Mannheim, Germany) were added to the 1 mL buffy coat-GEB mixture followed by digestion for 90 min at 56°C under constant shaking at 1,000 rpm. Eventually, DNA was extracted from 240 μL of this mixture and stored at −20°C until use. DNA concentrations were measured in the Nanodrop ND-1000 UV–vis spectrophotometer (NanoDrop Technologies, Wilmington, USA) or the Qubit 2.0 Fluorometer (Life Technologies, Carlsbad, USA).

### PCR

All PCR amplifications were carried out in 200 μL thin-wall PCR tubes (ABgene, Epsom, UK) in a T3 thermocycler 48 (Biometra, Göttingen, Germany). Amplified products were visualised under UV after electrophoresis in a 2% agarose gel at 135 V for 30 minutes and staining with ethidium bromide. To check the quality of DNA, a PCR targeting vertebrate cytochrome b was performed [72,73]. To detect *T. evansi* type A, the RoTat 1.2 PCR was conducted [37] while the EVAB PCR was used for the detection of *T. evansi* type B [34]. Detection of *T. vivax* was performed by means of *Tv*PRAC PCR [56]. ITS1-PCR was used to test part of the specimen collection for *T. congolense*, *T. theileri* and *Trypanozoon* [55]. Each PCR assay was done in 25 μL reaction volumes with 12.5 μL HotStarTaq polymerase master mix (Qiagen, Leipzig, Germany) containing 2.5 units HotStarTaq DNA polymerase, 1 × PCR buffer with 1.5 mM MgCl_2_ and 200 μM of each dNTP, 0.8 μM of each primer (Biolegio, Amsterdam, Netherlands), 8 μL accugene water (Lonza, Verviers, Belgium) and 2.5 μL of template DNA.

The target genes, primer names and sequences and expected amplicon lengths are represented in Table 1. Compared to the references cited in the table, some minor changes were made at the level of the polymerase and master mix, the initial denaturation step and the numbers of cycles. Cycling conditions for the different PCRs were as follows. Cytochrome B PCR: 94°C for 15 min and 35 cycles of 30 sec at 94°C, 30 sec at 52°C, 30 sec at 72°C and final extension for 5 min at 72°C. RoTat 1.2 PCR: 94°C for 15 min and 40 cycles of 30 sec at 94°C, 30 sec at 59°C, 30 sec at 72°C and final extension for 5 min at 72°C. EVAB PCR: 94°C for 15 min and 30 cycles of 30 sec at 94°C, 30 sec at 60°C, 60 sec at 72°C and final extension for 1 min at 72°C. TvPRAC PCR: 94°C for 15 min and 30 cycles of 30 sec at 94°C, 30 sec at 63°C, 30 sec at 72°C and final extension for 5 min at 72°C. ITS-1 PCR: 94°C for 15 min and 40 cycles of 30 sec at 94°C, 30 sec at 60°C, 30 sec 72°C and final extension for 5 min at 72°C.

**Table 1:** **Specifications of the PCR assays used in the study**

**Taxon**	**Target gene**	**Primers**	**Primer sequences**	**Amplicon length**	**Reference**
Vertebrates	Cytochrome b	L14841	5′-CCATCCAACATCTCAGCATGATGAAA-3′	400 bp	Adapted from [73]
		H15149	5′-GCCCCTCAGAATGATATTTGTCCTCA-3′		
*T. evansi* Type A	VSG RoTat 1.2	RoTat1.2-F	5′-GCGGGGTGTTTAAAGCAATA-3′	205 bp	Adapted from [37]
		RoTat1.2-R	5′-ATTAGTGCTGCGTGTGTTCG-3′		
*T. evansi* Type B	minicircle	EVAB-1	5′-ACAGTCCGAGAGATAGAG-3′	436 bp	Adapted from [34]
		EVAB-2	5′-CTGTACTCTACATCTACCTC-3′		
*T. vivax*	Proline racemase	*Tv*PRAC-F	5′ CGCAAGTGGACCGTTCGCCT- 3′	239 bp	Adapted from [56]
		*Tv*PRAC-R	5′ ACGCGGGGCGAACAGAAGTG-3′		
Diverse *Trypanosoma species*	ITS-1	ITS-1 F	5′-TGTAGGTGAACCTGCAGCTGGATC-3′	*T. vivax* 150 bp, *T. theileri* 350 bp, *Trypanozoon* 450 bp, *T. congolense* 650 bp	[44]
		ITS-1 R	5′-CCAAGTCATCCATCGCGACACGTT- 3′		

bp: base pairs.

### Data analysis

All data were recorded in Microsoft Excel. STATA/MP 13.1 [74] was used for statistical analysis. Percentages with 95% confidence interval (CI) were used to express prevalence. Logistic regression was applied for assessing differences in prevalence of AAT between domestic animal species and evaluating the effect of infection (test positive) on PCV values. To assess agreement between the diagnostic tests, Cohen’s kappa coefficient was calculated and interpreted according to Landis and Koch [75]. P-values <0.05 were considered as significant.

## Results

In total, 1811 animals were sampled of which 959 (53%) in Tigray and 852 (47%) in Afar. In general, there was statistically significant interaction (*X*^2^ = 330.12, p < 0.001) between regions and sampled domestic animal species, i.e. more cattle and camels were sampled in Tigray than in Afar, while more sheep and goats were sampled in Afar than in Tigray.

### Parasite detection

In 68 animals, trypanosomes were detected (Table 2). Thus, the overall parasitologically confirmed prevalence of trypanosomosis was 3.8% (CI 2.9-4.6%) with 4.7% (CI 3.4-6.0%) in Tigray and 2.7% (CI 1.6-3.8%) in Afar. No trypanosomes were detected in equines. The parasitological prevalence in cattle (7.3%, CI 5.0-9.5%) was significantly higher (z = 2.53, p = 0.011) than in camels (4.0%, CI 2.6-5.4%), sheep (0.6%, CI 0–1.7%) and goats (0.4%, CI 0–1.2%).

**Table 2:** **Test positives over total number of animals for each host species within each region**

**Diagnostic test**	**Region**	**Host species**						
		**Cattle**	**Camel**	**Goat**	**Sheep**	**Mule**	**Horse**	**Donkey**
mHCT	Tigray	32/411	11/343	1/60	1/64	0/10	0/25	0/46
	Afar	4/82	19/411	0/204	0/117	-	-	0/38
	**Total**	**36/493**	**30/754**	**1/264**	**1/181**	**0/10**	**0/25**	**0/84**
CATT/*T.evansi*	Tigray	169/411	39/343	12/60	14/64	0/10	0/25	2/46
	Afar	15/82	64/411	23/204	9/117	-	-	7/38
	**Total**	**184/493**	**103/754**	**35/264**	**23/181**	**0/10**	**0/25**	**9/84**
ITL	Tigray	0/411	21/343	1/60	1/64	0/10	0/25	0/46
	Afar	0/82	9/411	1/204	0/117	-	-	1/38
	**Total**	**0/493**	**30/754**	**2/264**	**1/181**	**0/10**	**0/25**	**1/84**
RoTat 1.2 PCR	Tigray	23/411	33/343	6/60	4/64	1/10	7/25	3/46
	Afar	7/82	55/411	4/204	0/117	-	-	2/38
	**Total**	**30/493**	**88/754**	**10/264**	**4/181**	**1/10**	**7/25**	**5/84**
EVAB PCR	Tigray	0/411	0/343	0/60	0/64	0/10	0/25	0/46
	Afar	0/82	4/411	0/204	0/117	-	-	0/38
	**Total**	**0/493**	**4/754**	**0/264**	**0/181**	**0/10**	**0/25**	**0/84**
*Tv*PRAC PCR	Tigray	13/411	16/343	2/60	1/64	0/10	0/25	0/46
	Afar	0/82	10/411	3/204	3/117	-	-	3/38
	**Total**	**13/493**	**26/754**	**8/264**	**4/181**	**0/10**	**0/25**	**3/84**

### Serology

With CATT/*T.evansi*, antibodies were detected in 354 animals (Table 2). Thus, the overall seroprevalence was 19.6% (CI 17.7-21.4%) with 24.6% (CI 21.9-27.3%) in Tigray and 13.9% (CI 11.5-16.2%) in Afar. Among the equines, *CATT/T.evansi* was only positive in donkeys (10.7%, CI 4.0-17.4%). The overall seroprevalence was significantly higher (z = 9.39, p < 0.001) in cattle (37.3%, CI 33.1-41.6%) than in camels (13.7%, CI 11.2-16.1%), in goats (13.3%, CI 9.2-17.4%), in sheep (12.7%, CI 7.8-17.6%) and in donkeys (10.7%, CI 4.1-17.4%).

With the ITL (Table 2), *T. evansi*-specific antibodies were detected only in 34 animals (30 camels, 2 goats, 1 sheep and 1 donkey). Thus, the seroprevalence in ITL was 1.9% (34/1811, CI 1.3-2.5%).

Kappa statistics indicated a poor but significant agreement between CATT/*T.evansi* and ITL (p < 0.001, Table 3).

**Table 3:** **Degree of agreement between diagnostic tests**

**Cross test**	**Observed (%)**	**Expected by chance (%)**	**Kappa**	**Z**	**p**
CATT/*T.evansi* and ITL	81.45	79.31	0.10	8.45	<0.001
CATT/*T.evansi* and RoTat 1.2 PCR	80.29	75.58	0.19	9.31	<0.001
RoTat 1.2 PCR and ITL	92.10	90.42	0.176	9.75	<0.001

### Molecular diagnosis

The overall molecular prevalence of *T. evansi* type A assessed with RoTat 1.2 PCR was 145/1811 or 8.0% (CI 6.8-9.3%) with 8.0% (CI 6.3-9.8%) in Tigray and 8.0% (CI 6.2-9.8%) in Afar (Table 2). The molecular prevalence of *T. evansi* type A in camels (11.7%, CI 9.4-14.0%) was significantly higher (p < 0.001) than in cattle (6.1%, CI 4.0-8.2%), donkeys (6.0%, CI 0.9-11.0%), goats (3.8%, CI 1.5-6.1%), and sheep (2.2%, 0.1-4.4%). The molecular prevalence of *T. evansi* type A was 28.0% (CI 10.4-45.6%) in horse and 10.0% (CI 7.6-27.6%) in mule. Kappa statistics indicated a poor but significant agreement between RoTat 1.2 PCR and the antibody detection tests, ITL and CATT/*T. evansi* (p < 0.001, Table 3). Among the 145 RoTat 1.2 PCR positives, only 71 were positive in CATT/*T.evansi* and only 18 were positive in ITL. Four camels, all from Awash Fentale district in Afar, were found positive in EVAB PCR indicating the presence of *T. evansi* type B. All four were negative in CATT/*T.evansi* and ITL although one of them was also positive in RoTat 1.2 PCR suggesting a mixed infection.

The overall molecular prevalence of *T. vivax* assessed with *Tv*PRAC PCR was 54/1811 or 3.0% (CI 2.2-3.8%) with 3.3% (CI 2.2-4.5%) in Tigray and 2.6% (CI 1.5-3.7%) in Afar (Table 2). The molecular prevalences of *T. vivax* were 3.5% (CI 2.2-4.8%) in camels, 3.0% in goats (CI 1.0-5.1%), 2.6% (CI 1.2-4.1%) in cattle and 2.2% (CI 0.1-4.4%) in sheep and were not significantly different (p = 0.925). All horses and mules were negative in *Tv*PRAC PCR. The molecular prevalence of *T. vivax* in cattle from Tigray was 3.2% (13/411) but was 0% in Afar. Among the 54 *Tv*PRAC PCR positives, 10 were also positive in CATT/*T.evansi* but were negative in RoTat 1.2 PCR. Only two camels and one goat were positive in both *Tv*PRAC PCR and RoTat 1.2 PCR.

Among the 68 parasitologically positive animals, 32 cattle, 1 camel and 1 sheep were negative in the RoTat 1.2 PCR, EVAB PCR and *Tv*PRAC PCR. To check for the possibility that mHCT detected *T. theileri* and *T. congolense*, ITS1-PCR was run on their specimens. Four cattle were positive for *T. vivax* and two cattle specimens were positive for *T. theileri*. Ten were negative. No single one was positive for *T. congolense*. Eighteen cattle specimens showed a profile with amplicons of different lengths that could not be interpreted unequivocally.

Among the CATT/*T.evansi* positive animals, 269 (77%) were negative in all PCR tests (165 cattle, 42 camels, 33 goats, 22 sheep and 7 donkeys).

### Packed cell volume (PCV)

In Table 4, the average PCV values and standard deviations (SD) are given according to the status of the animals in the mHCT, CATT/*T. evansi*, RoTat 1.2 PCR and *Tv*PRAC PCR. Camels that were found positive in those tests had a significantly lower average PCV than the animals that were negative in the different tests. The average PCV in ITL positive camels (24.2% ± 3.4%) was not significantly different from ITL negatives (25.7% ± 3.59%) (p = 0.05). In cattle and equines, the average PCV value was significantly lower only in CATT/*T.evansi* positive animals. In sheep and goats, no significant differences in average PCV were observed.

**Table 4:** **Average PCV of the animals according to their status in the different diagnostic tests**

**Test**	**Species**	**% PCV non-infected ± SD** ^**a**^	**% PCV infected ± SD** ^**a**^	**Regression coefficient value**	**t** ^**b**^	**P** ^**c**^
mHCT	Camels	25.8 ± 3.53	21.5 ± 2.53	−4.23	−6.50	<0.001*
	Cattle	25.9 ± 5.25	24.9 ± 5.49	−0.97	−1.07	0.287
CATT/*T.evansi*	Camels	25.9 ± 3.46	23.8 ± 3.87	−2.09	−5.59	<0.001*
	Cattle	26.6 ± 5.69	24.6 ± 4.19	−2.02	−4.20	<0.001*
	Equines	33.6 ± 6.3	27.9 ± 7.9	−5.71	−2.56	0.012*
	Goats	26.7 ± 5.84	24.9 ± 4.57	−1.79	−1.73	0.084
	Sheep	25.1 ± 5.57	22.9 ± 6.11	−2.12	−1.77	0.088
RoTat 1.2 PCR	Camels	25.0 ± 3.49	23.7 ± 3.81	−2.16	−5.39	<0.001*
	Cattle	25.8 ± 5.25	26.3 ± 5.56	0.53	0.54	0.591
	Equines	33.2 ± 6.6	33.1 ± 7.1	−0.98	−0.05	0.960
	Goats	26.5 ± 5.68	23.3 ± 5.89	−3.29	−1.79	0.074
	Sheep	24.8 ± 5.71	25.5 ± 3.89	0.74	0.26	0.796
*Tv*PRAC	Camels	25.7 ± 3.57	23.8 ± 3.77	−1.89	−2.65	0.008*
	Cattle	25.9 ± 5.30	23.1 ± 2.91	−2.83	−1.92	0.056
	Equines	33.2 ± 6.6	33.2 ± 5	−0.003	0.00	0.999
	Goats	26.4 ± 5.71	26.9 ± 6.30	0.543	0.26	0.792
	Sheep	24.8 ± 5.70	23.8 ± 4.33	−1.05	−0.36	0.716

^a^SD: standard deviation.

^b^t: Student’s t distribution value.

^c^P: probability.

*Statistically significant reduction in PCV.

## Discussion

In this cross sectional study, the mHCT, CATT/*T.evansi*, RoTat 1.2 ITL and RoTat 1.2 PCR, EVAB PCR and *Tv*PRAC PCR were used to assess the non-tsetse transmitted AAT prevalence in domestic animals in two regions of northern Ethiopia, Tigray and Afar. The overall prevalence of AAT as assessed with mHCT was 3.75% which was similar to AAT prevalence reported in cattle from other tsetse-free areas in Ethiopia (3.2% in Gondar and Bale Lowlands) using the same technique [44]. This is probably underestimating the real prevalence since mHCT is acknowledged to detect <50% of infections with low parasitaemia [49,76]. Although only one goat and one sheep were positive in mHCT, this finding confirms the presence of trypanosomosis in small ruminants [38,77-79]. The fact that no single equine was positive in mHCT while some of them were positive in the *T. evansi* specific RoTat 1.2 PCR and the *T. vivax* specific *Tv*PRAC PCR, indicates that in these animals the parasitaemia level remained under the lower detection limit of mHCT (about 60 trypanosomes/mL, [80].

With RoTat 1.2 PCR, it was confirmed that all domestic animals are susceptible to infection with *T. evansi* type A but that camels and horses are particularly at risk [21,22]. With EVAB PCR, we report for the first time the presence of *T. evansi* type B in camel in Ethiopia. Till today, *T. evansi* type B has only been isolated from camel in Kenya although indirect evidence exists that it also circulates in Sudan [31,32,81,82]. Furthermore, Hagos et al. suggested the existence of non-RoTat 1.2 *T. evansi* in camels from Bale zone in Ethiopia based on their finding that about one third of parasitologically positive camels were negative in CATT/*T.evansi* [45]. Also in our study, all four camels with *T. evansi* type B were negative in CATT/*T. evansi*. These data suggest that *T. evansi* type B is not confined to Kenya but may occur in more East African countries and even beyond, thus calling for the adaptation of serological and molecular diagnostic tests, like CATT/*T.evansi* and RoTat 1.2 PCR, to ensure detection of both types of *T. evansi* without compromising specificity.

Our study also confirms that *T. vivax* can infect diverse domestic animal species, including donkeys [4]. The overall molecular prevalence of *T. vivax* as assessed with *Tv*PRAC PCR was lower than reported in other studies [44,56]. The present study shows that camels in Ethiopia can be infected with *T. vivax* and that infection is associated with morbidity reflected by a significant reduction in PCV. Co-infections with *T. vivax* and *T. evansi* were rare (2 camels, 1 goat) but characterised by low PCV (20–22.5%). Mixed infection by both parasites was also reported in [83].

As expected, ITS1 PCR confirmed the absence of *T. congolense* in the mHCT positive animals that were negative in RoTat 1.2 PCR and *Tv*PRAC PCR but revealed four *T. vivax* infections that were not picked up by *Tv*PRAC PCR. Interestingly, ten mHCT positive animals remained negative in all PCRs. In the single sheep, the presence of the non-pathogenic *T. melophagium* cannot be ruled out but the other nine negatives remain unexplained [84,85]. Also unexplained remain the 18 cattle specimens showing a complex amplicon profile in ITS1 PCR, including a putative *T. vivax* specific 150 bp amplicon. In a previous study, which led to the development of the *Tv*PRAC PCR, we observed that the ITS1 PCR can generate non-specific amplicons in the presence of cattle DNA rendering unequivocal interpretation of the results impossible [44]. Although the analytical sensitivity of *Tv*PRAC is lower than of ITS1 PCR, it is still much higher than of mHCT [56]. Therefore, mHCT positive and *Tv*PRAC negative but ITS1 *T. vivax* positive specimens may be due to particular *T. vivax* strains not detectable in *Tv*PRAC. To further investigate these unexplained results, it will be necessary to isolate the trypanosomes detected in the mHCT, which will be particularly challenging in case of *T. vivax*. Indeed, *T. vivax* is notoriously difficult to grow in laboratory rodents and/or in culture [86,87].

Seroprevalence, as assessed with CATT/*T.evansi* was much higher than molecular prevalence which is not unexpected for several reasons. First, CATT/*T.evansi* cannot distinguish current from cured infection as detectable level of antibodies can persist for 2.3–22.6 month after trypanocidal treatment [88,89]. Secondly, in particular in chronic infections, parasitaemia can be well below the detection limit of parasitological and even molecular diagnostic tests, a phenomenon well known in human African trypanosomosis but less studied in AAT [90,91]. Finally, as CATT/*T.evansi* is not 100% specific, false positive cases do occur [92].

Still, the poor agreement between CATT/*T.evansi* and ITL is puzzling. Both serological tests detect antibodies against the VSG RoTat 1.2 that is considered specific for *T. evansi* type A. Although a limited loss in sensitivity of ITL when performed on filter paper eluates may be expected other factors may cause this discrepancy [70,93]. While ITL detects exclusively variant specific antibodies, CATT/*T.evansi* detects also antibodies directed against non-variant specific epitopes of VSG RoTAt 1.2 and other surface exposed antigens. Thus, infection with other trypanosomes, e.g. *T. vivax*, may lead to a positive result in CATT/*T.evansi* as was suggested in a study on bovine trypanosomosis in Suriname [47,60,94]. This cross-reactivity caused by *T. vivax* infection may explain why all CATT/*T.evansi* positive cattle specimens remained negative in ITL. However, it provides no explanation why the 30 cattle specimens that were positive in RoTat 1.2 PCR remained negative in ITL and why from the 145 RoTat 1.2 PCR positives, only 71 were also positive in CATT/*T.evansi*. Is it possible that the target sequence of RoTat 1.2 PCR is also present in some particular *T. vivax* strains circulating in Afar and Tigray but that the gene containing that sequence is a pseudogene or a gene that is not expressed during an infection? As we were not able to isolate *T. vivax* strains during this study, a conclusive answer to this question cannot be given.

If one considers a low PCV as a morbidity marker, it is striking that mainly camels are susceptible to AAT as disease. Indeed, camels that were positive in mHCT, CATT/*T.evansi*, RoTat 1.2 PCR and TvPRAC PCR had a significantly lower PCV than camels that were negative in all these tests. Among the other host species, only cattle and equines that were positive in CATT/*T.evansi* had a significantly lower PCV than CATT/*T.evansi* negative animals again suggesting that most CATT/*T.evansi* positive animals were actually infected, whether with *T. evansi* or *T. vivax*.

Among the parasitologically positive animals, three quarter presented without or with only mild symptoms. As in the study region, it is common to treat only sick camels and bovines with trypanocidal drugs such as diminazine and isometamidum, asymptomatic infections remain without treatment and constitute an uncontrolled reservoir for the disease.

Our study has some limitations. Although intended, it was not possible to compare the AAT prevalence between Tigray and Afar since the number of examined individuals per animal species was considerably different between two study regions. Also, no stained blood preparations were prepared that would have allowed morphological characterisation of those parasites that were detected in the mHCT but that remained negative in the species-specific PCRs.

## Conclusions

This study shows that non-tsetse transmitted AAT is an important threat to the health of camels, equine and ruminants in Afar and Tigray regions in Ethiopia. In these regions, AAT is caused by *T. vivax* and *T. evansi* type A and type B, the latter only in camels. Hence, improving serological and molecular diagnostic tests to detect both types of *T. evansi* as well as *T. vivax* is necessary. Unexplained results obtained with the current diagnostic tests in bovine specimens warrant particular efforts to isolate and characterise trypanosome strains that circulate in Northern Ethiopia.

## Abbreviations

CATT: Card agglutination test for trypanosomiasis
NTTAT: Non-tsetse transmitted animal trypanosomosis
RoTat: Rhode *Trypanozoon* antigenic type
*Tv*PRAC: *Trypanosoma vivax* proline racemase
VSG: Variant surface glycoprotein
